# Krukenberg tumor presenting as back pain and a positive urine pregnancy test: a case report and literature review

**DOI:** 10.1186/1757-2215-7-36

**Published:** 2014-04-07

**Authors:** Dalia Moghazy, Omar Al-Hendy, Ayman Al-Hendy

**Affiliations:** 1 Center for Women Health Research, Meharry Medical College, Nashville, Tennessee, USA; 2 Department of Obstetrics and Gynecology, Center for Women’s Health Research, Meharry Medical College, 1005 Dr. D.B. Todd Jr. Blvd., 37208 Nashville, Tennessee, USA

**Keywords:** Elevated β-hCG, Adenocarcinoma, Paraneoplastic syndrome, Krukenberg tumors

## Abstract

A Krukenberg tumor is a rare and potentially deadly cause of elevated serum β-hCG as part of a paraneoplastic syndrome. This study aims to describe the unusual case of a 36-year-old woman that presented to the Emergency Department (ED) with back pain and a positive urine pregnancy test. Assessment revealed no intrauterine pregnancy and a small left ovarian cyst. Further investigation showed moderately differentiated gastric adenocarcinoma with distant metastases to the spine. The patient died less than 3 months after her first presentation to the ED. Paraneoplastic syndrome, albeit rare, should be considered in the differential diagnosis of elevated β-hCG due to the high mortality associated with Krukenberg tumors.

## Background

Krukenberg tumor is an ovarian adenocarcinoma metastasis from a primary malignancy of the gastrointestinal tract with 76% originating from the stomach [1]. It is bilateral in 80% of the cases [1]. The eponym comes from the description of 5 cases by Friedrich Krukenberg (1871–1946) in 1896 [1]. He described it as common in young women, presenting with ascites, uneven knobby ovarian surfaces, and lymphatic involvement [1]. These characteristics are still applicable today. When a woman of reproductive age presents to the Emergency Department (ED) with abdominal, pelvic, or back pain, a urine β-human chorionic gonadotropin (β-hCG) pregnancy test is typically and appropriately part of the initial laboratory work up. If positive, the woman is presumed pregnant until proven otherwise. If an intrauterine pregnancy is ruled out, the search continues for a source of the elevated β-hCG. The differential diagnosis includes ectopic pregnancy, germ cell ovarian tumors, gestational trophoblastic neoplasia including hydatiform mole and placental site trophoblastic tumors [2], phantom hCG resulting from interference in serum testing [2], and paraneoplastic syndrome as a rare subset of adenocarcinomas that secrete β-hCG. Malignancies that produce β-hCG may originate from various locations such as gastric mucosa, lung, colon, cervix, and endometrial areas [3]. A gastric origin is the most frequent, ranging from 11% to 17% of this rare subset [3].

## Case

We present the case of a 36 year old G_4_P_4_ Hispanic female who presented to the ED for nausea, vomiting, diarrhea, 30 pound weight loss over 2–3 months, generalized body aches most pronounced in the lower back for 5 days, and a positive pregnancy test. Past medical history was significant for hypertension, dysphagia, 4 caesarean sections and bilateral tubal ligation. Family history was noncontributory. She denied tobacco, alcohol, or intravenous drug abuse. Physical exam revealed reproducible pain upon palpation of the spine. The bimanual exam was negative for discharge, cervical motion tenderness, or pelvic tenderness. A routine urine pregnancy test was positive and subsequent serum testing demonstrated a β-hCG level of 154 mIU/ml. The patient denied sexual intercourse in over 2 years and confirmed having a normal menstrual period less than 2 weeks prior. The patient was admitted to the hospital for further assessment and management. Initial abdominal and transvaginal ultrasound examination revealed no intrauterine pregnancy, non-visualization of the right ovary, and a complex cyst measuring 3.5 cm in diameter on the left ovary. Her repeat serum β-hCG 48 hours later was 155 mIU/ml demonstrating an insignificant change from the earlier reading. A Computed tomography (CT) scan with contrast of the thorax showed clear lungs and a cluster of enlarged lymph nodes in the celiac axis, with the largest being 1 cm. The patient was consented for laparoscopic evaluation, bilateral salpingectomy, possible left ovarian cystectomy, peritoneal washings and dilatation and curettage. At time of surgery, pelvic washings were collected and they were negative for malignant cells. Review of the pelvis revealed normal anatomy. The cyst on the left ovary was removed and sent for frozen section pathology that revealed a hemorrhagic corpus luteal cyst. The uterine curretings were also sent for frozen section pathology that showed late secretory endometrium and no chorionic villi. The patient had a normal postoperative course and was discharged from the hospital on the first postoperative day.

**Figure 1:**
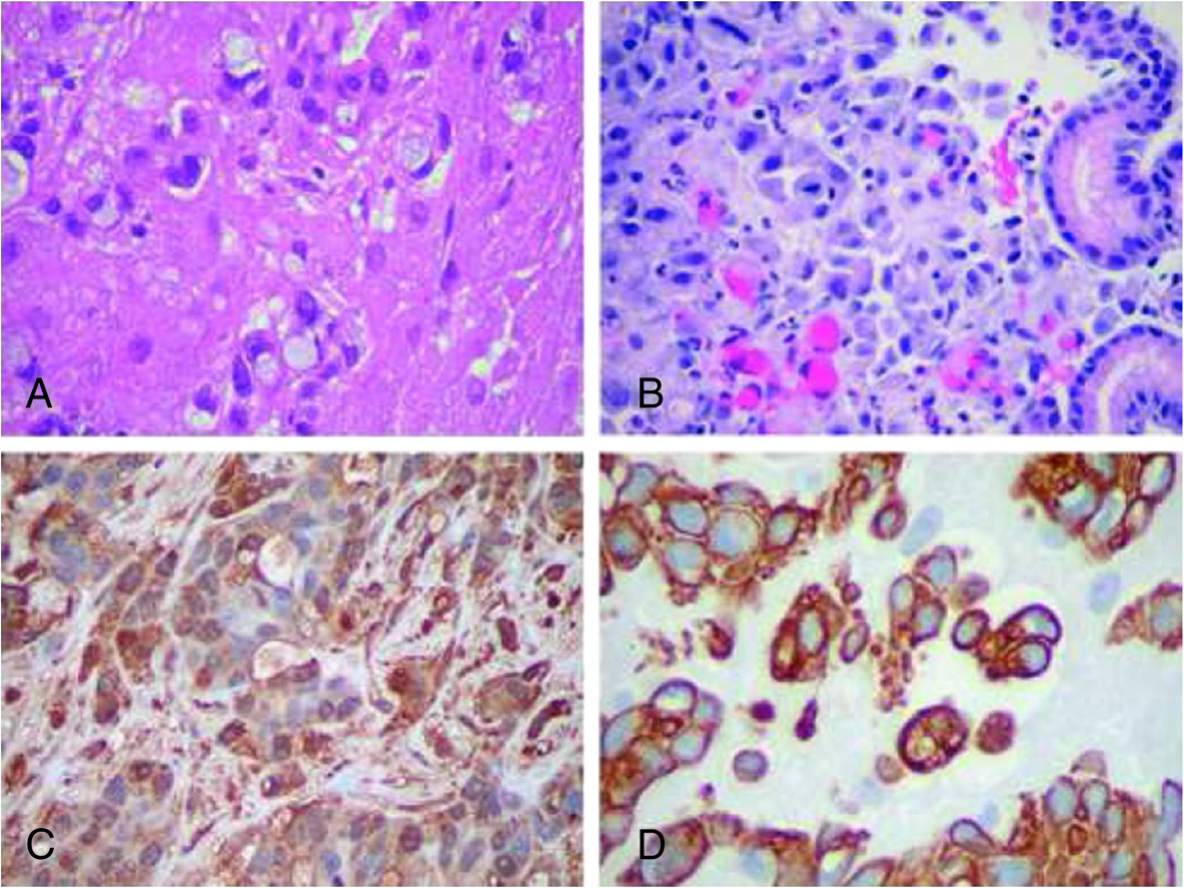
**Microscopic appearance of resected tumor. (A)** Left ovary: Numerous signet-ring cells with pale cytoplasm are distributed singly or in nests in a luteinized stroma (hematoxylin-eosin stain, ×400). **(B)** Gastric biopsy: The lamina propria contains individually infiltrating, discohesive, poorly differentiated adenocarcinoma cells (hematoxylin-eosin stain, ×400). **(C)** Immunostain for β-hCG demonstrates strong diffuse cytoplasmic and membranous staining within the malignant cells (immunoperoxidase technique with DAB chromogen, ×400). **(D)** Immunostain for cytokeratin 7: The malignant cells are strongly immunopositive (immunoperoxidase technique with DAB chromogen, ×400) cell tissue from left ovarian cyst wall.

Upon discharge, her serum β-hCG level was 175 mIU/ml. The final pathology report on the left corpus luteum cyst located a small focus of moderately differentiated adenocarcinoma in corpus luteum staining strongly positive for Cytokeratin 7 (CK7) and negative for Cytokeratin 20 (CK 20) suggesting a primary gastric or pancreatic carcinoma. The patient was referred to the oncology service. Magnetic resonance imaging (MRI) of the pelvis/abdomen demonstrated left para-aortic and paraceliac adenopathy, likely metastatic in nature. No discrete masses were seen in the ovaries. Bone scan showed enhancement in the right pedicle of L3 vertebra highly suspicious for metastasis. At this point, the patient reported increasing abdominal and back pain. Her laboratory investigation was significant for CA-125 of 134 U/ml (reference range: 0.0-21.0), CEA level of 37.2 ng/ml (reference range: 0.0-10.0), and CA 19–9 3230 U/ml (reference range: 0–37). Computed tomography (CT) guided fine needle aspiration and core biopsy of the L3 vertebral body were positive for malignant cells of poorly differentiated metastatic carcinoma, strongly positive for β-hCG, α-inhibin, and human placental lactogen (HPL). Multiple biopsies collected at colonoscopy and esophagogastroduodenoscopy showed a poorly differentiated adenocarcinoma in the lamina propria of the stomach fundus positive for AEI/3, CD7, CK20 and CEA (Figure 1). The esophageal biopsy showed atypical cells with necrotic exudates and the colon biopsy showed no malignancy. The patient underwent 2 weeks of radiation therapy on the L3 lesion and was readmitted to hospital for pain control and supportive services for her bilateral lower extremity weakness.

**Figure 2:**
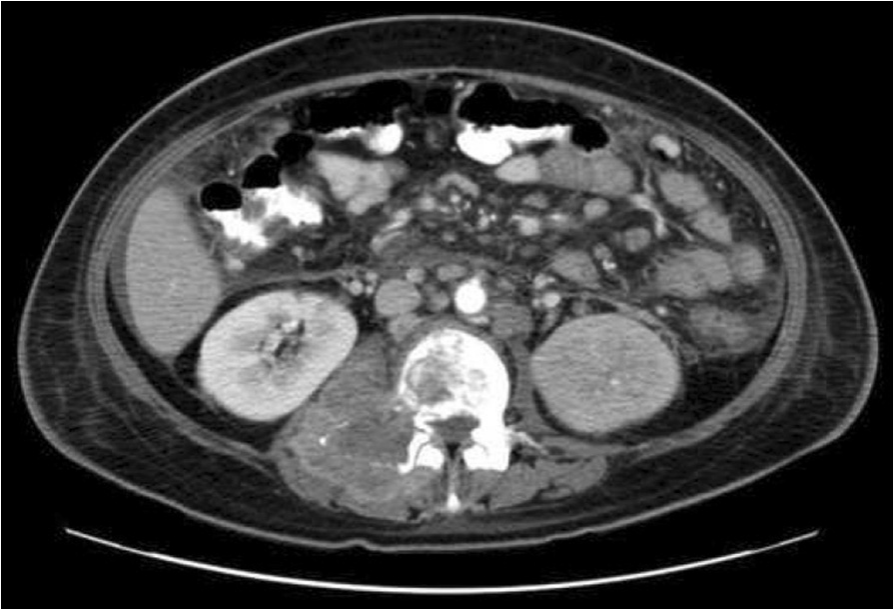
Computed tomography of the abdomen/pelvis with contrast showing a heterogenous 4-cm mass involving the body and pedicle of the L3 vertebra on the right; there is severe loss of vertebral body height and retropulsion of bony fragments into the spinal canal, creating severe stenosis.

Therapeutic intervention with chemotherapy was contraindicated as the patient was found to have urosepsis. A repeat CT abdomen/pelvis showed widely metastatic gastric adenocarcinoma with possible compression of cauda equina at L3 and metastases to the liver, spleen, left adrenal, and left acetabulum (Figure 2). In addition, the CT image revealed peritoneal carcinomatosis, retroperitoneal and mesenteric adenopathy, left hydronephrosis secondary to compression by metastatic adenopathy, acute pancreatitis and splenic vein thrombosis. During hospitalization the patient expired secondary to urosepsis, disseminated intravascular coagulation, and widespread metastatic disease.

## Discussion

Krukenberg tumor is a rare metastatic signet ring cell tumor of the ovaries that accounts for 1-2% of all ovarian tumors [4]. The stomach is the primary site of metastasis for the majority of these tumors via retrograde lymphatic spread [4]. Patients with Krukenberg tumor tend to be young, with an average age of 45 years old. Patients usually present with symptoms related to ovarian involvement such as abdominal pain and distention [4]. The remainder of patients present with nonspecific gastrointestinal complaints or are asymptomatic. Ascites is usually present in 50% of cases and usually reveal malignant cells [4]. The primary tumor is usually too small to detect and calls for careful radiographic and endoscopic exploration [4]. Abdominal and pelvic ultrasonography and CT usually reveal bilateral, solid ovarian masses but cystic masses can also occur.

In this case, the patient presented with non-specific gastrointestinal complaints such as nausea, vomiting, diarrhea, low back pain, and a 30 pound weight loss over 2–3 months. The patient had a positive β-hCG despite having a normal menstrual period 2 weeks prior and lack of sexual intercourse in over two years as well as a prior bilateral tubal ligation. Typically, after a positive urine pregnancy test, a rise in serum β-hCG of >66% in 48 hours is highly suggestive of intrauterine pregnancy [5]. Transvaginal ultrasound is used to confirm the presence of an intrauterine pregnancy when the β-hCG is greater than 1500 mIU/ml. However, in this case, the patient’s β-hCG was low at 154 mIU/ml and remained relatively unchanged after 48 hours at 155 mIU/ml. If the ß-hCG level plateaus or fails to double in 48 hours and no intrauterine pregnancy is detected via imaging techniques, an ectopic pregnancy is highly suspected. In this patient, abdominal and transvaginal ultrasound revealed no intrauterine pregnancy and a 3.5 cm complex cyst of the right ovary. A low β-hCG in a patient can possibly indicate an early normal intrauterine pregnancy which usually complicates the management option and argues for expectant management. However the lack of normal rise in β-hCG in this case ruled out that possibility.

β-hCG is a hormone glycoprotein composed of an alpha and beta subunit. β-hCG is normally produced by placental syncytotrophoblast however, it can also be produced by other tissues such as the testis, liver, lung, colon, and stomach [6]. Therefore a tumor in any of these tissues may lead to an elevated β-hCG level. Another rare cause of elevated serum hCG is the phenomenon of phantom hCG or phantom choriocarcinoma. This occurs when there are persistent low levels of hCG found in the serum without evidence of a pregnancy, tumor, or trophoblastic disease. Phantom hCG is due to a substance in the serum such as heterophilic antibodies that interfere with the hCG immunoassay [7]. Urine pregnancy test is typically negative in this situation as these heterophilic antibodies are too large pass the kidney glomerular membrane. Determining the true cause of the elevated hCG is critical for appropriate intervention and can prevent unnecessary and possibly harmful management such as possible chemotherapy for putative choriocarcinoma [7].

## Conclusion

Physicians should be aware that elevated β-hCG can be found in conditions outside of pregnancy and can be associated with malignancy. When persistently low levels of β-hCG are detected, a Krukenberg tumor should be considered in the differential diagnosis after excluding more common etiologies. Only 25-30% of patients with Krukenberg tumor have a primary malignancy when the Krukenberg tumor is found [1]. Detailed imaging studies of the abdomen and pelvis should be performed to search for a primary malignancy. The prognosis of a Krukenberg tumor is poor with a median survival rate of 14 months [4] since such metastasis signifies rapid cell growth and proliferation. The prognosis is poor if the primary tumor is found after metastasis to the ovaries and even worse if the primary tumor remains undetected [4]. There is no established optimal treatment for Krukenberg tumor. However, studies using aggressive targeted chemotherapy and surgical debulking are potentially promising [3].

## Consent

All patient information used in the manuscript were de-identified and anonymised therefore no consent form was necessary.

## Abbreviations

ED: Emergency Department; hCG: Human chorionic gonadotropin; CT: Computed tomography; MRI: Magnetic resonance imaging; CK7: Cytokeratin 7; CK 20: Cytokeratin 20; HPL: Human placental lactogen.

## Competing interests

The authors declare that they have no competing interests.

## Authors’ contributions

DM drafted the manuscript and was the corresponding author. AAH followed the patient during hospitalization and performed the surgical intervention and has given final approval of the version to be published. OAH has performed edited the manuscript for language and grammar. All authors have read and approved the final manuscript.
